# 한국 산모의 산후 우울과 산후 외상 후 스트레스장애 관련성: 종단적 연구

**DOI:** 10.4069/kjwhn.2022.02.18

**Published:** 2022-03-30

**Authors:** Hyunjin Cho, Minseon Koh, Hyeji Yoo, Sukhee Ahn

**Affiliations:** 1 College of Nursing, Chungnam National University, Daejeon, Korea; 1 충남대학교 간호대학; 2 Department of Nursing, Yeoju Institute of Technology, Yeoju, Korea; 2 여주대학교 간호학부

**Keywords:** Postpartum depression, Post-traumatic stress disorders, Social support, Spouse, 산후 우울, 외상 후 스트레스 장애, 사회적 지지, 배우자

## Abstract

**Purpose:**

This study aimed to determine the level of postpartum posttraumatic stress disorder (PTSD) and postpartum depression (PPD) in Korean mothers with healthy babies and to explore the factors related to postpartum PTSD.

**Methods:**

This study used a longitudinal survey design to explore the levels and association of PPD and PTSD. Two hundred women were recruited during pregnancy and the data were collected via online survey from 166 mothers (84% retained) who gave birth to healthy babies, at two postpartum periods: Fear of childbirth was assessed at the 1st week; and spousal support, PPD, and postpartum PTSD were surveyed at the 4th week postpartum. Descriptive statistics, t-test, one-way analysis of variance, Chi square test, and multiple regression were done.

**Results:**

The mean age of mothers was 33.12 (±3.97) years old. Postpartum PTSD was low (8.95±6.49) with 1.8% (n=3) at risk (≥19). PPD was also low (6.68±5.28) and 30.1% (n=50) were identified at risk (≥10). The comorbid rate of PPD with PTSD was 6%. Mothers who did not have a planned pregnancy had higher scores of PPD (t=–2.78, *p*=.008), whereas spousal support and PPD had negative relationship (r=–.21, *p*=.006). The overall explanatory power for postpartum PTSD was 55.2%, of which PPD was the only significant variable (β=.76, t=13.76, *p*<.001).

**Conclusion:**

While only 1.8% was at risk of postpartum PTSD at 4 weeks postpartum, PPD prevalence was 30.1% and PPD was the only influential factor of postpartum PTSD. Assessment and counseling of PPD are required as well as screening for postpartum PTSD. More research is also needed on postpartum PTSD in Korean women.

## Introduction

여성은 임신과 출산으로 다양한 생리적, 정서적 변화를 경험하는데, 특히 출산을 경험한 후 산후 우울이나 산후 외상 후 스트레스장애(posttraumatic stress disorder, 이후 산후 PTSD라 칭함)를 경험할 수 있다[1]. 산후 우울은 산후 4주 이내, 전반적인 정신 및 행동의 변화가 나타나는 시기의 우울한 기분, 극도의 불안, 불면증 등을 동반하는 기분 저하 증상으로[2], 흔히 우울 증상으로 나타난다. 산후 우울의 유병률은 국외 연구의 메타 분석에서 산모의 5%–20%로[3], 국내에서는 18%–19%로 나타났다[4]. 산후 PTSD는 2008년에 문제가 지적되면서 연구가 시작되었으며, 출산 과정에서 신체 및 정서적인 위협이나 손상에 직면하거나 노출된 경험 및 그로 인한 두려움, 무력감, 공포감을 느끼는 사건을 의미한다[5]. 실제로 산후 PTSD를 경험한 산모의 질적 연구의 메타 합성 결과, 산후 PTSD는 ‘출산 후 강한 부정적 감정, 바람직한 자신과 가족에 대한 상실감, 산산이 부서져버린 관계’로 확인되었다[6]. 산후 PTSD 발생률은 국외 연구에서 고위험 임신과 출산을 경험하지 않은 산모 중 3.1%–4.0%로[6,7], 국내에서는 만삭으로 출산하여 건강한 신생아를 키우는 산모 중 7.5%로[8] 나타나 국내에서 발생률이 더 높다. 산후 PTSD의 발생 시기는 산후 4–6주이며 6개월에 2.6%, 12개월에 2.4%로, 최대 12개월까지 증상이 지속함을 알 수 있다[9]. 산후 PTSD를 적절하게 진단 및 치료하지 않으면 산후 모유 수유율이 낮아지고 모아 애착관계 형성이 안되어 적응 장애가 나타날 수 있다. 이는 후속 임신에서의 재발로 이어질 수도 있으며[10], 장기적으로 여성의 삶에 부정적인 영향을 끼친다[11]. 이에 출산을 경험하는 산모를 대상으로 산후 PTSD 수준을 확인하고 관련 요인을 탐색하여 산모와 가족의 정신건강 관리를 위한 근거와 중재 전략으로 활용할 필요가 있다.

과거 PTSD 경험, 임신 중 분만 두려움, 출산 시 난산이나 충격적인 출산경험, 잘못된 대처 및, 스트레스, 산후 우울, 산후 건강문제가 산후 PTSD를 증가시키는 유의한 영향요인으로 나타났다[6,7,12]. 특히 산후 우울은 산후 PTSD와 높은 상관을 갖고 있으며[1,13,14], 산후 PTSD는 산후 우울을 동반한다[15,16]. 즉 산후 3개월째이면서 산후 PTSD를 가진 산모 대부분이 산후 우울의 가능성을 내포하고 있고, 출산경험이 생각날 때마다 산후 PTSD와 우울 증상이 동시에 나타나고 있었다[16]. 그러나 산후 PTSD와 우울 모두 부정적인 기분 상태를 포함하기에 산후 PTSD의 증상은 산후 우울 증상으로 쉽게 오인될 수 있다고 지적하였다[1]. 그러나 국내에서는 산모의 산후 우울 발생률이나 영향요인 연구가 다수 수행된 것에 비해, 산후 우울과 관련 있는 산후 PTSD 발생률 또는 두 요인 간 관련성을 탐색하는 연구는 한국판 도구개발 연구 두 편이 유일하다[8,17]. 이중 산후 1개월된 산모를 대상으로 수행한 도구개발 연구에서는 산후 PTSD가 산후 우울과 높은 상관을 보였고, 산후 우울이 산후 PTSD에 유의한 영향을 미친 것으로 나타났다[8]. 생후 1–18개월 된 아기를 키우는 산모에게 도구 타당화 검사를 한 연구에서도 산후 PTSD가 우울, 불안과 상관이 높게 나타났다[17]. 두 연구의 제한점은 미숙아 또는 건강한 신생아를 둔 산모 자료를 통합한 보고라는 점으로, 건강한 신생아를 출산한 산모에서의 산후 PTSD와 우울 간 관련성은 아직 확인되지 않은 상태이다. 따라서 건강한 산모를 대상으로 산후 우울과 PTSD를 동시에 사정하여 산후의 우울과 PTSD 발생률과 더불어 산후 우울 및 PTSD 동반 발생률을 확인할 필요가 있다.

산후 PTSD 관련 요인으로 산후 우울과 더불어 부정적인 출산경험, 계획하지 않은 임신, 제왕절개 분만, 그리고 산후 사회적 지지를 탐색하고자 한다. 산모의 출산경험의 긍정적 측면으로 모성기 전환에서 개인의 성장과 회복력 증가를 들 수 있지만, 출산 시 경험한 두려움은 부정적인 출산경험으로 이어지면서 산후 PTSD를 높이는 예측요인이다[6,13,18]. 국내 임부를 대상으로 분만 두려움을 사정한 결과 26.5%가 심각한 분만 두려움을 갖고 있었고, 분만 두려움은 산전 우울과 양의 상관을 보였다[19]. 나아가 임신 중 분만 두려움, 출산 시 심한 통증, 통제력 부족, 출산경험의 부정적 인식은 산후 PTSD 발생에 유의한 상관성과 영향력을 나타내었다[1,6,13,20]. 또한 계획하지 않은 임신은 산후 우울의 발생 위험을 높이는 요소였다[21-23]. 분만 유형에서는 질 분만 대비 제왕절개 분만을 경험한 산모에서 산후 우울 위험이 63% 증가하였고[24] 기계보조 질 분만 또는 응급제왕절개 분만이 PTSD의 위험요소[1]로 확인되었지만, 국내 연구에서는 산모의 제왕절개 수술 여부는 위험요소로서 유의성이 없었다[8].

출산 후 부모기 적응에 있어 배우자의 신체적, 심리적 지지는 산모의 신체 및 정서 회복에 매우 중요하기 때문에, 배우자의 지지 부족은 산후 우울을 설명하는 주요 요인이다[21,25]. 그러나 주산기에 배우자 지지가 부족하고 배우자간 부정적인 감정이 해결되지 못하면 산후 우울, 불안 및 초기 PTSD 증상으로 이어질 수 있다[1,7,25,26]. 실제로 배우자의 지지가 부족한 여성에서의 산후 PTSD 비율은 지지를 받는 여성에 비해 3배 높게 나타났다[27]. 이는 배우자의 지지 역할이 출산 후 관리에서 잠재적인 긍정적 요소로 중요하기 때문에 산후 PTSD 감소와 관련이 있음을 시사한다.

최근에는 산후 PTSD의 발생률과 관련 요인 및 결과에 대한 연구들이 주로 미국, 유럽의 저위험 또는 고위험 임산부들을 대상으로 보고되고 있고[7,10], 국내에서는 산후 PTSD 측정을 위한 도구개발 연구만 보고된 상태이다[8,17]. 우리나라 저위험 임신과 출산을 경험하는 여성에서 주산기 우울 발생률이 증가하고 있지만[28], 산후 우울이나 산후 PTSD 관련 건강문제를 사정하고 진단 및 치료를 받는 과정에서 종종 여성이 배제되거나 과소평가되고 있는 것이 현실이다[29]. 이에 본 연구에서는 산모의 산후 PTSD와 산후 우울 수준을 확인하고 산후 PTSD 관련 요인을 탐색하여 추후 산모의 정신건강 위험 상태의 조기 선별, 산후 정신건강 증진과 출산 준비를 위한 산전교육 프로그램에 근거를 제공하고자 한다.

본 연구는 산모의 산후 우울과 산후 PTSD의 출산 시 경험한 두려움과 배우지 지지 수준, 계획하지 않은 임신, 제왕절개 분만관련성을 확인하기 위함이며, 구체적인 연구 목적은 다음과 같다.

(1) 산후 우울, 산후 PTSD, 출산 시 경험한 두려움, 배우자 지지의 수준을 확인한다.

(2) 산모의 일반적/산과적 특성에 따른 산후 우울과 산후 PTSD의 차이를 검정한다.

(3) 산후 우울, 산후 PTSD, 출산 시 경험한 두려움, 배우자 지지의 관련성을 탐색한다.

(4) 산후 우울, 출산 시 경험한 두려움, 배우자 지지, 계획하지 않은 임신, 제왕절개 분만이 산후 PTSD에 미치는 영향을 확인한다.

## Methods

Ethics Statement: This study was approved by the Institutional Review Board of Chungnam National University (201904-SB-044-01). Informed consent was obtained from the participants.

### 연구 설계

본 연구는 산모의 산후 우울과 산후 PTSD의 관련성을 탐색하고 산후 PTSD의 관련 요인(산후 우울, 출산 시 경험한 두려움, 배우자 지지)을 규명하기 위한 상관성 조사연구 설계이다. 본 연구는 임신기부터 산후까지 우울과 분만 두려움 관련 요인을 탐색하고자 산전에서 산후 4주까지 3회에 걸쳐 시행한 종단적 조사연구(longitudinal study)의 일부 자료를 사용하였다. 임신기에 수집한 1차 조사자료는 임부의 분만 두려움 관련 요인 탐색 연구[19]한 것으로, 본 연구에서는 이에 대해 산후 2회 추적조사를 통해 수집한 산모 자료를 이용하였다. 본 연구는 STROBE 보고지침(https://www.strobe-statement.org)에 따라 기술하였다.

### 연구 대상

연구 대상은 대전 지역에 소재한 2개의 여성전문병원에서 건강한 만삭아를 출산하고 직접 돌보는 산모 166명이다. 제외 기준은 보건복지부가 지정한 11대 고위험 임신질환 진단[4]을 받은 경험이 있거나, 출산한 신생아가 사망하였거나, 또는 아기가 신생아집중치료실에 입원해 있는 산모이다.

선행연구[13]에서 산후 우울은 산후 PTSD에 대해 46%의 설명력을 보였다. 본 연구가 국내 산모에서 산후 우울이 산후 PTSD에 미치는 영향력을 처음 탐색하는 것이기에 회귀분석에 사용할 효과크기는 f^2^=.10로 설정하였다. 이에 G*power 프로그램을 사용하여 유의수준 (α)=0.05, 검정력(1–β)=0.8, 효과크기 f^2^=.10, 예측요인 5개를 이용하여 표본크기를 계산한 결과, 최소 134명의 표본이 필요하였다. 1차 조사에 참여한 임부 200명을 대상으로 출산 후에 추적 조사를 통해 산후 1주와 1개월째 자료를 수집한 결과, 최종 대상자는 166명의 산모였고 출산 후 연구 참여율은 84%로 나타났다.

### 연구 도구

본 연구에서 사용한 도구는 연구팀이 원 도구의 개발자에게 연락을 취하여 도구 사용에 대한 승낙을 받았다.

#### 산후 우울

산후 우울은 Edinburgh Postnatal Depression Scale [29]에 대한 한국어판 산후우울 도구[30]를 이용하여 산후 1개월째 측정하였다. 이 도구는 우울, 불안 및 공포, 죄책감, 자해 사고 등에 대한 자가 보고형의 10문항, 4점 척도로, 각 문항은 “전혀 그렇지 않다” 0점부터 “대부분 그렇다” 3점으로 구성된다. 1, 2, 4번 문항을 제외한 나머지 문항은 역 채점하고 총점은 30점이며, 점수가 높을수록 우울한 것으로 평가한다. 국내의 산모를 대상으로 절단점을 9/10로 한 연구[30]를 근거로 하여 본 연구에서도 절단점을 9/10로 사용하였다. 원 도구의 신뢰도는 Cronbach’s α=.82 [29], 한국판 도구에서는 .84 [30], 본 연구에서는 .85였다.

#### 산후 PTSD

본 연구에서는 Callahan 등[31]이 5점 척도로 수정한 산후 PTSD 도구에 대한 한국어판 주산기 PTSD 도구[8]를 이용하여 산후 1개월째에 측정하였다. 이 도구는 총 14문항의 5점 척도로, 하위 영역은 아기의 입원에 대한 악몽 등 외상의 재경험(1–3번 문항), 회피 행동(4–9번 문항), 과각성 및 죄책감(10–14번 문항)으로 구성된다. 각 문항은 “전혀 그렇지 않다” 0점부터 “한 달 이상 자주 그렇다” 4점으로 구성된다. 총점 56점 중에서 19점 이상이면 임상적으로 유의한 PTSD 증상을 보인다고 판단한다. 5점 척도로 수정한 원 도구의 신뢰도는 .90 [31], 한국판 도구에서는 .87 [8], 본 연구에서는 .76으로 나타났다.

#### 출산 시 경험한 두려움

출산 시 경험한 두려움은 Wijma 등의 산후 분만 두려움 측정 도구인 Wijma Delivery Experience Questionnaire - version B[32]의 한국판 분만 두려움 도구[33]를 이용하여 산후 1주일 시점에 측정하였다. 이 변수의 측정 시점을 산후 1개월이 아닌 1주로 설정한 이유는 출산 시 경험한 두려움에 대한 생각과 감정이 시간이 갈수록 감소하기 때문에 출산 시 감정을 가능한 빨리 반영하기 위함이다. 이 도구는 33문항의 6점 척도로, “매우 그렇다” 0점부터 “전혀 그렇지 않다” 5점으로 구성된다. 총점은 165점으로 점수가 높을수록 출산 시 경험한 두려움이 더 강하다는 것을 의미한다. 절단점은 85점 이상으로, 분만 두려움의 정도를 두려움과 극심한 두려움으로 보았다. 원 도구의 신뢰도는 Cronbach’s α=.86 [32], 한국판 도구에서는 .90 [33], 본 연구에서는 .89였다.

#### 배우자 지지

배우자 지지는 배우자가 산후 관리와 아기 돌봄을 통해 산모를 지지한 정도를 측정하는 도구[34]를 이용하여 산후 1개월 시점에 측정하였다. 이 도구는 총 13문항의 4점 척도로 “전혀 수행하지 않음” 1점부터 “열심히 수행함” 4점으로 구성된다. 총점은 13–52점으로 점수가 높을수록 배우자 지지가 높음을 의미한다. 원 도구의 신뢰도는 Cronbach’s α=.94[34], 본 연구에서는 .89로 나타났다.

#### 산모의 일반적, 산과적 특성

산후 1주 시점에 산모의 산과적 특성으로 분만 형태(질 분만, 제왕절개 분만), 아기의 성별, 재태기간, 출생 시 체중, 산모의 주관적 건강상태(좋음, 좋지 않음)에 대해 조사하였다. 산모의 일반적 특성(나이, 직업, 학력, 가정경제 상태, 산과력, 계획임신 여부)은 임신기 설문 조사에 응답한 자료를 사용하였다.

### 자료 수집

본 연구는 대전 지역에 소재한 2개의 여성전문병원의 병원장과 간호부장에게 연구의 목적과 방법을 설명하고 연구 진행에 대한 허락을 받았다. 자료 수집은 2019년 6월 16일부터 2020년 2월 1일까지 시행하였다. 연구자는 연구 모집 공고문을 병원 외래에 게시하였고, 공고문을 보고 자율적으로 참여를 원하는 임부들을 대상으로 연구의 필요성 및 목적을 설명하고 연구 참여를 원하는 임부에게 연구 참여에 대한 서면 동의를 얻었다. 연구에 자발적 참여와 중도 탈락의 자유를 소개하고 사생활 보호 및 비밀 유지에 대해 설명하였다. 연구 참여에 동의한 임부들은 산전 관련요인 및 일반적 특성 설문지를 작성하였다. 연구자는 대상자의 추적 조사용 전화번호를 통해 산후조사 시점을 문자로 알릴 것을 사전에 설명하였다. 출산 후 발송한 문자에 응답한 산모에게는 산후 조사를 위한 온라인 설문조사 링크를 발송하였다. 산후 1주에는 산모의 산과적 특성과 출산 시 경험한 두려움을 측정하고 산후 1개월에는 산후 우울, 산후 PTSD, 배우자 지지를 측정하였다. 설문에 소요된 시간은 측정 시점마다 평균 10–15분 내외였다. 설문지 작성을 완료한 대상자에게 소정의 답례품을 조사 시점별로 제공하였다.

### 자료 분석 방법

수집된 자료는 IBM SPSS ver. 24.0 for Windows (IBM Corp., Armonk, NY, USA)를 이용하여 분석하였으며 통계적 유의 수준은 α= 0.05로 설정하였다.

(1) 대상자의 일반적, 산과적 특성, 산후 우울, 산후 PTSD, 출산 시 경험한 두려움, 배우자 지지에 대한 수준은 빈도분석과 기술통계를 이용하여 분석하였다.

(2) 대상자 일반적, 산과적 특성에 따라 산후 우울과 산후 PTSD에 대한 차이 검정은 t-test, 일원분산분석(one-way analysis of variance), 또는 카이제곱검정, 필요 시 Fisher exact test로 분석하였다.

(3) 출산 시 경험한 두려움, 산후 우울과 산후 PTSD 발생률의 관계는 카이제곱검정(Fisher exact test)로 분석하였고, 출산 시 경험한 두려움, 배우자 지지, 산후 우울, 산후 PTSD의 관련성은 상관관계 분석을 통해 분석하였다.

(4) 산후 PTSD의 영향요인은 다중회귀분석을 통해 분석하였다.

## Results

### 대상자의 일반적 특성과 산과적 특성

산모의 나이는 평균 33.12세(standard deviation [SD], 3.97세)로 35세 미만인 여성은 111명(66.9%)이었다. 직업이 없는 산모는 89명(53.6%), 대학교 졸업 이상의 학력 소지자는 152명(91.6%), 200만 원–399만 원의 월수입을 보고한 자는 89명(53.6%)이었다. 산과적 특성으로 첫 출산을 경험한 산모는 68명(41.1%), 계획임신을 한 산모는 56명(33.7%)이고, 질 분만을 한 산모는 131명(78.9%)이었다. 아기의 성별은 남아가 102명(61.4%)였고, 출생아의 평균 체중은 3.28 kg (SD, ±0.32 kg), 평균 재태기간은 37.6주(SD, ±1.22주)였다(Table 1).

### 산후 우울, 산후 PTSD, 출산 시 경험한 두려움, 배우자 지지의 수준

산후 우울은 30점 만점에 평균 6.68점(SD, 5.28점)으로 낮은 수준을 보였고, 10점 이상의 산후 우울증 위험군은 50명(30.1%)으로 나타났다. 산후 PTSD는 56점 만점에 평균 8.95점(SD, 6.49점)을 보여 낮은 수준을 보였고, 임상적으로 유의한 PTSD 증상을 보인 위험군은 절단점 19점 이상을 기준으로 평가할 때 3명(1.8%)으로 나타났다. 출산 시 경험한 두려움은 165점 만점에 평균 65.28점(SD, 16.03점)으로 보통 수준을 보였고, 절단점 85점을 기준으로 평가할 때 심각한 수준의 두려움은 22명(13.2%)에서 나타났다. 배우자 지지는 13–52점 기준에 평균 41.82점(SD, 7.96점)으로 높은 수준의 지지를 보였다(Table 2).

### 대상자의 일반적 특성과 산과적 특성에 따른 산후 우울과 산후 PTSD 차이

대상자의 특성 중에서 계획임신 여부는 산후 우울(t=-2.78, *p*=.008)에 유의한 차이를 보였다. 즉 임신을 계획하지 않은 산모는 임신을 계획한 산모에 비해 산후 우울 점수가 유의하게 높게 나타났다(Table 1).

### 산후 우울, 산후 PTSD, 출산 시 경험한 두려움, 배우자 지지 간 관계

#### 산후 우울, 산후 PTSD, 출산 시 경험한 심각한 두려움 발생률 간 관계

심각한 출산 두려움을 경험한 여성의 59.1%가 산후 우울군에 속한 반면, 중등도 이하의 두려움을 경험한 여성의 25.7%가 산후 우울군에 속하였다. Fisher exact test로 변수 간 관련성을 검정한 결과, 심각한 출산 두려움 경험과 산후 우울은 유의한 관련성을 나타냈다(*p*=.006). 심각한 출산 두려움을 경험한 여성 중 9.1%가 산후 PTSD를 경험한 반면, 중등도 이하의 두려움이 있는 경우에는 0.7%의 여성이 산후 PTSD를 경험하였다. 검정 결과 심각한 출산 두려움과 산후 PTSD는 유의한 관련성을 보였다(*p*=.002). 산후 우울 위험군 50명(30.1%) 중에서 산후 PTSD 위험군에 속하는 여성은 3명(6.0%)으로 나타난 반면, 산후 우울이 없는 산모에서는 산후 PTSD가 한 건도 발생하지 않았다. 검정 결과 산후 우울과 산후 PTSD 간 유의한 관련성을 확인하였다(*p*=.026) (Table 3).

#### 산후 우울, 산후 PTSD, 출산시 경험한 두려움, 배우자 지지간 관계

상관분석을 통해 연구 변수 간 관련성을 탐색한 결과, 산후 PTSD는 산후 우울과 높은 양의 상관(r=.75, *p*<.001)을 보였고, 산후 우울은 배우자 지지(r=–.21, *p*=.006)와 약한 음의 상관을 보였다(Table 4).

### 산후 PTSD에 영향을 미치는 요인

문헌고찰에서 산후 PTSD에 영향을 보인 계획임신 여부, 출산 방법, 배우자 지지, 출산 시 경험한 두려움, 산후 우울을 독립변수로 입력하여 산후 PTSD에 영향을 미치는 요인을 탐색하였다. 회귀식의 가정(정규성, 선형성 다중공선성)과 잔차 진단(잔차의 정규성, 오차의 독립성, 등분산성)을 통해 자료의 적합도를 확인하였다. Durbin-Watson 값이 1.88으로 기준 2에 가까워 오차의 독립성을 확보하였다. 독립변수의 다중 공선성은 공차 한계와 분산팽창계수로 확인한 결과, 공차 한계 값은 0.77–0.99, 분산팽창계수 값은 1.00–1.29의 범위에 있어 다중공선성의 문제가 없는 것으로 나타났다. 회귀식의 적합도를 검정한 결과, 회귀식이 유의하였고(F=41.60, *p*<.001) 설명력은 55.2%로 나타났다. 독립변수 중 산후 우울이 유의한 설명요인으로 나타났다(β=.76, t=13.76, *p*<.001). 즉 산후 우울 점수가 높을수록 산후 PTSD 점수가 상승하였다(Table 5).

## Discussion

산후 1개월째인 산모의 산후 우울 발생률(절단점 10점 기준)은 30.1%로 나타났다. 이는 동일 도구와 절단점으로 평가한 종단적 연구에서 보고한 산후 2주째(32.0%)와 6주째(23.6%) [28], 산후 6주째인 초산모의 유병률(14.6%) [35]과 비교 시 높은 비율이다. 또한 국외 연구의 메타 분석에서 산후 1–6주 산모에게 동일 도구의 절단점 13점으로 평가한 유병률(9.3%–18.5%) [3]보다 높다. 이는 산후 우울을 측정한 산후 2–6주 시점이 대부분의 산모가 가정에서 신생아 양육을 직접 수행하는 시기이고, 초산 여부와 산후 문화에 따라 산욕기 모성 적응을 위한 사회적 지지의 양이나 질이 줄어드는 시기라는 점이 우울 발생률에 영향을 미친 것으로 보인다[3,35,36].

산모의 산후 PTSD 점수는 8.95로 낮은 수준이었다. 이는 산후 4–6주에 건강한 만삭아를 키우는 산모가 보고한 점수(9.10) [8]와 비교 시 약간 낮은 편이다. 반면 국외 연구에서 보고한 점수(8.25) [13]보다는 높게 나타났지만, 이런 점수 차이는 대상자의 인종이나 출산 및 산후 관리에 대한 문화적 차이 때문이라고 본다. 산모의 1.8%는 임상적으로 의미 있는 PTSD군으로 분류되었다. 이는 건강한 만삭아를 출산한 지 1개월째 된 우리나라 산모에서의 발생률(7.5%) [8]과 비교 시 낮은 비율이다. 국외 연구에서 출산 관련 PTSD 발생률은 메타분석 결과 3.1% [6], 산후 2개월째 독일인 산모에서 2.3% [35], 산후 6–8주째 중국인 산모에서 6.1% [37]로 각기 다르게 나타났다. 그 이유는 산후 PTSD 측정 시점이 연구마다 다른 것과, 인종‧지역 및 사회문화적 특성에 따라 출산 환경에 차이가 있기 때문으로 보인다[24,27].

산후 우울군과 산후 PTSD군에 모두 속하는 여성은 6.0%로 나타났다. 이는 미국인 산모에서 산후 우울과 산후 PTSD가 동반된 비율(15.7%) [16]과 비교 시 낮은 수준이다. 그 이유는 본 연구의 경우 산후 1개월째에 측정한 반면, 국외 연구에서는 산후 6개월째에 장기 효과를 측정하였기 때문으로 보인다. 본 연구에서 확인한 심각한 출산 두려움 경험, 산후 우울 및 산후 PTSD 발생의 관련성은 출산에 대한 심각한 두려움을 경험한 경우 산후 PTSD 점수가 높게 나타난 연구[18]와 부정적 출산경험이 산후 PTSD에 유의한 영향을 미친 연구[13,27,35]를 지지하였다. 이는 산모의 정신건강을 사정할 때 출산 시 경험한 두려움을 산후 우울과 산후 PTSD와 더불어 평가하는 것이 중요함을 시사한다.

배우자의 지지와 산후 우울의 부적 상관은 배우자의 지지가 높을 때 산후 우울에 보호효과가 있다는 연구[21,25]와 일치하였다. 반면 산후 PTSD에는 배우자 지지와의 상관성이나 영향력이 없어, 배우자의 지지 감소가 산후 스트레스와 우울증을 증가시켜 산후 PTSD와 같은 정신장애로 발전할 수 있다는 연구[15-16,37]와 약간 차이가 있다. 산후 사회적 지지의 구조나 기능 측면을 모두 측정하여 산후 PTSD와의 관련성을 추가로 탐색할 필요가 있다.

제왕절개 분만은 산후 PTSD에 영향력을 나타내지 못하였다. 이는 제왕절개 분만 여부가 산후 PTSD와 관련이 없다는 연구[35,38]와 유사하다. 반면 계획하지 않은 응급 제왕절개 분만이 산후 PTSD 발생률을 높이는 데 영향을 미친 연구[27]와는 다른 결과이다. 본 연구에서는 분만 유형 자료를 질 분만 또는 제왕절개 분만으로만 수집하였기에, 제왕절개 분만 중 예상치 못한 응급 제왕절개 분만 시행 여부에 대한 자료를 수집하지 못하여 이를 반영할 수 없었다. 추후 연구에서 분만 유형별 세부 정보를 수집하여 그 관련성을 재탐구할 필요가 있다.

계획하지 않은 임신을 한 여성은 계획임신을 한 경우에 비해 산후 우울 점수가 유의하게 높았다. 계획하지 않은 임신을 산전 우울과 산후 우울 모두에 유의한 영향요인으로 보고한 선행연구와 일치한다[21-23]. 이는 산모 간호 시 계획임신 여부를 주요 변수로 사정하고, 계획하지 않은 임신을 한 산모에게 보다 세심한 관찰과 중재가 필요함을 시사한다. 반면 다중회귀분석에서는 계획하지 않은 임신이 산후 PTSD에 미치는 영향력이 없었다. 그 이유는 임신과 출산을 거치는 동안 계획하지 않은 임신보다는 출산경험과 산후 우울상태와 같은 최근의 감정 상태가 산후 PTSD에 더 강한 영향을 보이기 때문[16]이라 생각한다.

산후 PTSD는 산후 우울과 높은 상관을 보였고, 회귀분석에서 산후 우울이 산후 PTSD를 설명하는 강력한 영향요인으로 나타났다. 이는 산후 우울이 출산 후 PTSD를 유의하게 예측한 선행연구[13-16,37,38]와 일치한다. 이 결과는 산후 PTSD와 우울 모두 부정적인 기분 상태를 포함하기에 산후 PTSD의 증상을 산후 우울로 쉽게 오인할 수 있다는 선행연구 결과[1]를 지지하는 것이다. 이를 토대로 산후 우울과 산후 PTSD를 사정하고 두 가지 문제의 동반 발생을 경험하는 산모를 선별하는 간호 사정이 중요하다. 간호사는 산후 우울과 산후 PTSD 위험군을 조기 발견하여 이들을 정확한 진단 및 치료 단계로 연계할 수 있어야 할 것이다.

본 연구는 한 지역에서 편의 표집한 건강한 아기를 출산한 산모를 대상자로 하였기에, PTSD 발생률을 해석할 때 주의를 기울여야 한다. 추후 더 큰 표본 수를 확보하여 임신에서 산후까지 관련 요인을 고려하면서 산후 우울과 PTSD 관계를 확인하는 반복 연구가 필요하다. 그러나 본 연구는 건강한 신생아를 키우는 산모에서 산후 우울과 산후 PTSD 관련성이 높고, 산후 우울군 속에 산후 PTSD군이 6% 존재함을 확인함으로써 산후 우울을 가진 산모들에게 산후 PTSD도 함께 사정해야 하는 근거를 확보한 점에서 의의가 있다.

결론적으로 본 연구에 참여한 산모의 30.1%가 산후 우울을, 산모의 1.8%가 산후 PTSD를 나타냈고, 산후 PTSD의 유의한 영향요인은 산후 우울로 확인되었다. 본 연구는 정상 임신과 출산을 경험한 산모를 대상으로 산후 PTSD의 영향요인을 파악하였기에, 추후 연구에서는 고위험 임신을 경험한 산모를 대상으로 임신, 출산 및 산후 특성이 산후 우울과 산후 PTSD에 미치는 영향을 탐색할 필요가 있다. 또한 실무에서 출산 후 검진 목적으로 병원에 방문한 산모에게 산후 우울과 PTSD를 사정하고, 산후 우울과 PTSD의 예방과 조기 관리를 위한 정신건강 상담이나 중재 의뢰가 요구된다.

## Figures and Tables

**Table 1.** Differences in postpartum depression and PTSD by participant characteristics (N=166)

Variable	Categories	n (%) or mean±SD	Postpartum depression		Postpartum PTSD	
			Mean±SD	t/F (*p*)	Mean±SD	t/F (*p*)
Age (year)		33.12±3.97				
	<35	111 (66.9)	7.13±0.52	1.68 (.094)	9.54±6.55	1.41 (.160)
	≥35	55 (33.1)	5.76±0.61		8.03±6.37	
Employment status	Yes	77 (46.4)	6.75±5.47	0.16 (.870)	9.02±6.95	–0.04 (.968)
	No	89 (53.6)	6.61±5.14		9.06±6.15	
Level of education	Up to high school	14 (8.4)	5.14±3.99	–1.13 (.257)	8.57±6.90	–0.28 (.776)
	University or more	152 (91.6)	6.82±5.37		9.09±6.50	
Monthly family income (KRW)	<2 million	7 (4.2)	6.71±5.67	1.02 (.360)	7.85±7.38	0.59 (.551)
	2 million–3.99 million	89 (53.6)	6.14±5.08		8.65±6.52	
	≥4 million	70 (42.2)	7.35±5.49		9.67±6.45	
Parity	Primipara	68 (41.0)	6.32±5.32	–0.72 (.470)	8.73±6.68	–0.51 (.608)
	Multipara	98 (59.0)	6.92±6.92		9.26±6.42	
Planned pregnancy	Yes	56 (33.7)	5.17±4.70	–2.78 (.008)	7.67±6.80	–1.95 (.053)
	No	110 (66.3)	7.44±5.42		9.74±6.28	
Type of birth	Vaginal birth	131 (78.9)	6.67±5.20	–0.04 (.966)	8.95±6.45	–0.35 (.720)
	Cesarean birth	35 (21.1)	6.71±5.67		9.40±6.84	
Mothers’ perceived health	Healthy	161 (97.0)	6.60±5.24	–1.08 (.281)	9.07±6.51	0.29 (.769)
	Not healthy	5 (3.0)	9.20±6.72		8.20±7.29	
Infant’s sex	Male	102 (61.4)	6.62±5.18	–0.16 (.870)	9.09±6.64	0.12 (.901)
	Female	64 (38.6)	6.77±5.48		8.96±6.35	
Infant’s gestational age (week)		37.6±1.22				
Infant’s birth weight (kg)		3.28±0.32				

KRW: Korean won (1 million KRW is approximately 900 US dollars), PTSD: posttraumatic stress disorder.

**Table 2.** Levels of postpartum PTSD, postpartum depression, spousal support, and fear of childbirth experienced (N=166)

Variable	Categories	n (%) or mean±SD	Possible range	Data range
*At postpartum first week*				
Fear of childbirth experienced		65.28±16.03	0–165	33–111
	Severe (≥85)	22 (13.2)		
	Moderate (65–84)	67 (40.4)		
	Low (≤64)	77 (46.4)		
*At postpartum first month*				
Spousal support		41.82±7.96	13–52	17–52
		166 (100)		
Postpartum depression		6.68±5.28	0–30	0–19
	At risk (≥10)	50 (30.1)		
	Normal	115 (69.9)		
Postpartum PTSD		8.95±6.49	0–56	0–22
	At risk (≥19)	3 (1.8)		
	Normal	163 (98.2)		

PTSD: Posttraumatic stress disorder.

**Table 3.** Postpartum depression and postpartum PTSD according to fear of childbirth (N=166)

Variable	Postpartum depression, n (%)		*p*	Postpartum PTSD, n (%)		*p*
	Yes	No		Yes	No	
Fear of childbirth experienced						
Severe	13 (59.1)	9 (40.9)	.006^†^	2 (9.1)	20 (90.9)	.002^†^
Non-severe	37 (25.7)	107 (74.3)		1 (0.7)	143 (99.3)	
Postpartum depression						
At risk (>10)				3 (6.0)	47 (94.0)	.026^†^
No				0 (0)	116 (100)	

PTSD: Posttraumatic stress disorder.

† Fisher exact test.

**Table 4.** Relationships among postpartum PTSD, postpartum depression, fear of childbirth, spousal support, birth type and planning of pregnancy (N=166)

Variable	r (*p*)			
	Postpartum PTSD	Postpartum depression	Fear of childbirth experienced	Spousal support
Postpartum PTSD	1			
Postpartum depression	.75 (<.001)	1		
Fear of childbirth experienced	.14 (.071)	.19 (.017)	1	
Spousal support	–.11 (.142)	–.21 (.006)	.03 (.748)	1
Cesarean birth	.03 (.720)	.12 (.140)	.23 (.003)	–.07 (.406)
Unplanned pregnancy	.15 (.053)	.19 (.013)	.03 (.684)	.13 (.092)

PTSD: Posttraumatic stress disorder.

**Table 5.** Factors influencing postpartum posttraumatic stress disorder (N=166)

Factor	β	t	*p*
Postpartum depression	.76	13.76	<.001
Spousal support	.04	0.83	.405
Fear of childbirth experienced	.01	0.21	.832
Type of birth^†^	-.05	–1.09	.276
Planned pregnancy^†^	.01	0.15	.881
F(*p*)		41.60	<.001
	Adjusted R**²=**.552		

† The indicator groups were as follows: type of birth (cesarean birth) and planned pregnancy (no).

## References

[1] Ayers S, Bond R, Bertullies S, Wijma K (2016). The aetiology of post-traumatic stress following childbirth: a meta-analysis and theoretical framework. Psychol Med.

[2] Silverman ME, Reichenberg A, Savitz DA (2017). The risk factors for postpartum depression: a population-based study. Depress Anxiety.

[3] Liu X, Wang S, Wang G Prevalence and risk factors of postpartum depression in women: a systematic review and meta-analysis. J Clin Nurs.

[4] Hwang JY (2020). Reclassification of high-risk pregnancy for maternal-fetal healthcare providers. J Korean Soc Matern Child Health.

[5] Leeds L, Hargreaves I (2008). The psychological consequences of childbirth. J Reprod Infant Psychol.

[6] Grekin R, O'Hara MW (2014). Prevalence and risk factors of postpartum posttraumatic stress disorder: a meta-analysis. Clin Psychol Rev.

[7] Yildiz PD, Ayers S, Phillips L (2017). The prevalence of posttraumatic stress disorder in pregnancy and after birth: a systematic review and meta-analysis. J Affect Disord.

[8] Kang JH, Rha DW, Kwon JY, Kim TY, Kim KR, Lee S (2011). A study of reliability and validity on the Korean version of perinatal PTSD questionnaire. J Korean Soc Depress Bipolar Disord.

[9] Gankanda WI, Gunathilake IA, Kahawala NL, Ranaweera AK (2021). Prevalence and associated factors of post-traumatic stress disorder (PTSD) among a cohort of Srilankan post-partum mothers: a cross-sectional study. BMC Pregnancy Childbirth.

[10] Dikmen-Yildiz P, Ayers S, Phillips L (2018). Longitudinal trajectories of post-traumatic stress disorder (PTSD) after birth and associated risk factors. J Affect Disord.

[11] Cook N, Ayers S, Horsch A (2018). Maternal posttraumatic stress disorder during the perinatal period and child outcomes: a systematic review. J Affect Disord.

[12] Boorman RJ, Devilly GJ, Gamble J, Creedy DK, Fenwick J (2014). Childbirth and criteria for traumatic events. Midwifery.

[13] Çapik A, Durmaz H (2018). Fear of childbirth, postpartum depression, and birth-related variables as predictors of posttraumatic stress disorder after childbirth. Worldviews Evid Based Nurs.

[14] Thiel F, Dekel S (2020). Peritraumatic dissociation in childbirth-evoked posttraumatic stress and postpartum mental health. Arch Womens Ment Health.

[15] Agius A, Xuereb RB, Carrick-Sen D, Sultana R, Rankin J (2016). The co-existence of depression, anxiety and post-traumatic stress symptoms in the perinatal period: a systematic review. Midwifery.

[16] Dekel S, Ein-Dor T, Dishy GA, Mayopoulos PA (2020). Beyond postpartum depression: posttraumatic stress-depressive response following childbirth. Arch Womens Ment Health.

[17] Park YK, Ju HO, NA H (2016). Reliability and validity of the Korean version of the perinatal post-traumatic stress disorder questionnaire. J Korean Acd Nurs,.

[18] Garthus-Niegel S, von Soest T, Vollrath ME, Eberhard-Gran M (2013). The impact of subjective birth experiences on post-traumatic stress symptoms: a longitudinal study. Arch Womens Ment Health.

[19] Cho H, Ahn S (2020). Do childbirth confidence, prenatal depression, childbirth knowledge, and spousal support influence childbirth fear in pregnant women?. Korean J Women Health Nurs.

[20] Alipour Z, Lamyian M, Hajizadeh E (2012). Anxiety and fear of childbirth as predictors of postnatal depression in nulliparous women. Women Birth.

[21] Kızılırmak A, Calpbinici P, Tabakan G, Kartal B (2021). Correlation between postpartum depression and spousal support and factors affecting postpartum depression. Health Care Women Int.

[22] Salsabilla DA, Prasetya H, Murti B (2020). The effect of unplanned pregnancy on postpartum depression: a meta-analysis. J Matern Child Health.

[23] Beck CT Postpartum depression: a metaphorical analysis. J Am Psychiatr Nurses Assoc.

[24] Bell AF, Andersson E (2016). The birth experience and women's postnatal depression: a systematic review. Midwifery.

[25] Don BP, Mickelson KD (2012). Paternal postpartum depression: the role of maternal postpartum depression, spousal support, and relationship satisfaction. Couple Family Psychol,.

[26] Shlomi Polachek I, Huller Harari L, Baum M, Strous RD (2014). Postpartum anxiety in a cohort of women from the general population: Risk factors and association with depression during last week of pregnancy, postpartum depression and postpartum PTSD. Isr J Psychiatry Relat Sci.

[27] Orovou E, Dagla M, Iatrakis G, Lykeridou A, Tzavara C, Antoniou E (2020). Correlation between kind of cesarean section and posttraumatic stress disorder in Greek women. Int J Environ Res Public Health.

[28] Yoo H, Ahn S, Oh J, Park S, Kim J, Koh M (2021). Depression and stress in Korean parents: a cohort study. Appl Nurs Res.

[29] Cox JL, Holden JM, Sagovsky R (1987). Detection of postnatal depression. Development of the 10-item Edinburgh Postnatal Depression Scale. Br J Psychiatry.

[30] Kim YK, Hur JW, Kim KH, Oh KS, Shin YC (2008). Clinical application of Korean version of Edinburgh postnatal depression scale. J Korean Neuropsychiatr Assoc.

[31] Callahan JL, Borja SE, Hynan MT (2006). Modification of the Perinatal PTSD Questionnaire to enhance clinical utility. J Perinatol.

[32] Wijma K, Wijma B, Zar M (1998). Psychometric aspects of the W-DEQ; a new questionnaire for the measurement of fear of childbirth. J Psychosom Obstet Gynaecol.

[34] Yang J, Jung IS (2018). Convergence effect of spouse’s support on postpartum depression and self-efficacy in primipara. J Korea Converg Soc.

[35] Kress V, von Soest T, Kopp M, Wimberger P, Garthus-Niegel S (2021). Differential predictors of birth-related posttraumatic stress disorder symptoms in mothers and fathers - a longitudinal cohort study. J Affect Disord.

[36] Yoo H, Ahn S, Park S, Kim J, Oh J, Koh M (2021). Factors influencing prenatal and postpartum depression in Korea: a prospective cohort study. Korean J Women Health Nurs.

[37] Liu Y, Zhang L, Guo N, Jiang H (2021). Postpartum depression and postpartum post-traumatic stress disorder: prevalence and associated factors. BMC Psychiatry.

[38] Gankanda WI, Gunathilake IA, Kahawala NL, Ranaweera AKP (2021). Prevalence and associated factors of post-traumatic stress disorder (PTSD) among a cohort of Srilankan post-partum mothers: a cross-sectional study. BMC Pregnancy Childbirth.

